# *Steinernema carpocapsae* jumps with greater velocity and acceleration than previously reported

**DOI:** 10.17912/micropub.biology.000435

**Published:** 2021-08-02

**Authors:** Adler R Dillman, Wyatt Korff, Michael H Dickinson, Paul W Sternberg

**Affiliations:** 1 Department of Nematology, University of California, Riverside; 2 HHMI-Janelia Research Campus; 3 Division of Biology and Biological Engineering, Caltech

## Abstract

Infective juveniles of the insect-parastic nematode *Steinernema carpocapsae *can**jump greater than 6 times their height, a striking evolved novelty in some species of this genus. Using high-speed videography, we observed the kinematics of *Steinernema carpocapsae *spontaneous**jumping behavior. Our analysis places a lower bound on the velocity and acceleration of these worms.

**Figure 1:**
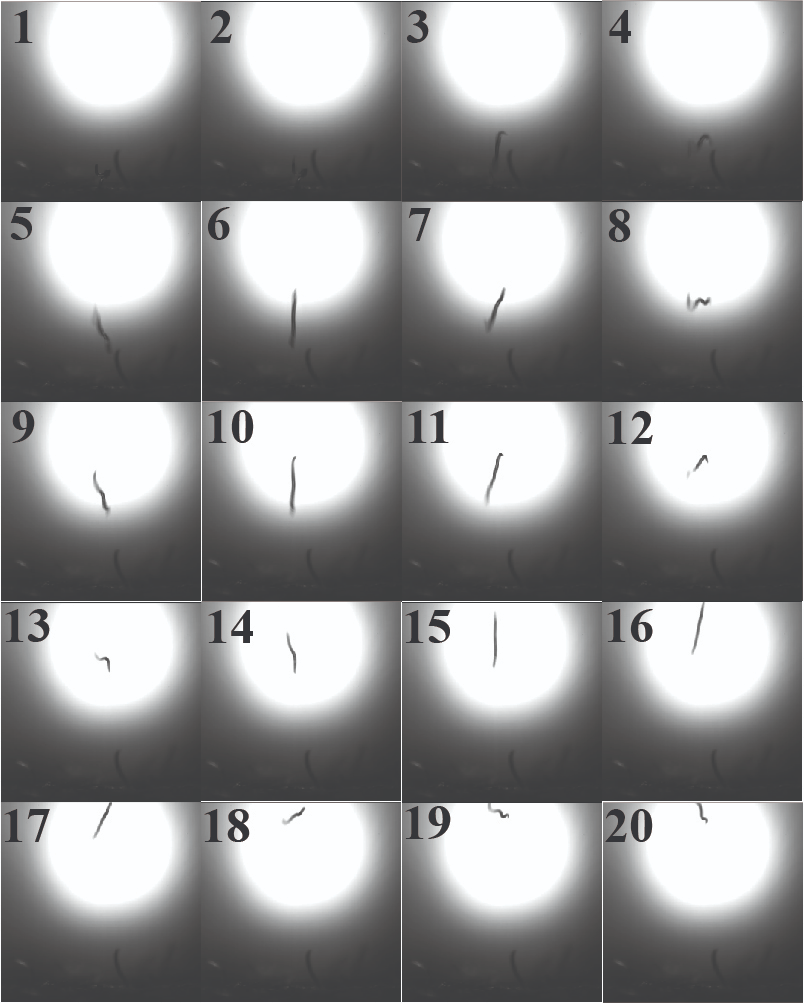
*Behavioral sequence for* S. carpocapsae *IJ jumping* (Video 1)*. The sequence shown, from image 1 to image 20, occurs in approximately 3.88 milliseconds.*

## Description

Many invertebrates are capable of jumping, such as locusts, fleas, mantids, and cockroaches. Some invertebrates can jump without the use of their legs, such as click beetles and springtails (Mo *et al.*, 2020). Nematodes, which are non-segmented roundworms, are capable of a variety of complex behaviors, though jumping is arguably the most visually striking. Jumping is a rare behavior among nematodes, with only a few species in the genus *Steinernema* being reported as capable of jumping (Campbell and Kaya, 1999). The kinematic performance of *S. carpocapsae* jumping was first described in 1999, with the peak velocity and peak acceleration reported as 1.13 m/s and 164 g, respectively (Campbell and Kaya, 1999). These data were not calculated based on direct observation and high-speed videography, but were estimated based on the distance, height, and timing of the nematode jumps. This nematode is reported to be capable of jumping a distance of 4.8 mm, and a height of 3.9 mm (Campbell and Kaya, 1999).

We investigated the kinematics of *Steinernema carpocapsae* jumping behavior using high-speed videography (Videos 1 & 2). We calculated peak velocity to be 1.9 m/s and peak acceleration to be 38205 m/s^2^ (3899 g), based on measurements from jumping sequences filmed at 20 kHz and using the average infective juvenile length of 558 µm (Nguyen *et al.*, 2007) (Video 2). The velocity calculated here is one and a half times greater and the acceleration is more than twenty times greater than what has been previously reported for *S. carpocapsae* jumping (Campbell and Kaya, 1999). The jumping acceleration of *S. carpocapsae* is significantly greater than fleas, click beetles, or any other jumping animal for which acceleration has been reported. These measurements are likely underestimates of both peak velocity and acceleration, as the jumping sequence probably needs to be recorded at 50 kHz or greater and captured from multiple angles to get more accurate measurements (deVries *et al.*, 2012).

## Methods

*Steinernema carpocapsae* (All) was reared in last instar waxworm larvae using standard methods (Kaya and Stock, 1997). Infections were performed in 6 cm Petri dishes where ~30 infective juveniles (IJs) per host were placed on filter paper. Infections were incubated at 23C for 7-10 days and then placed on White traps. IJs were collected, rinsed three times with tap water, and then placed in a cell culture flask. IJs were used for jumping within 10 days. To observe jumping behavior, IJs were placed on filter paper and a gentle puff of CO_2_ was delivered from above using a 10 ml Hamilton gastight syringe equipped with a Hamilton blunt needle (22s/2”/3) (Hallem *et al.*, 2011). Jumping was visualized using a Photron FastCam SA5 high-speed infrared-sensitive video camera (Photron, San Diego, CA, USA) with a Fostec 8375 fiber optic light source with an EKE bulb. Take-offs were filmed at 8,000 s^-1^ and 20,000 s^-1^ with 1024×1024 pixel resolution, using an InfiniVar video microscope lens (Infinity Photo-Optical, Centennial, CO, USA) (Card and Dickinson, 2008). The lens was set approximately 30 mm from the surface with the nematodes, positioned perpendicular to the plane of worm jumping. Sequences where animals moved out of plane were excluded. Filmed jumps were digitized and analyzed using custom Matlab software. Analysis was calibrated based on the reported average length of *S. carpocapsae* IJs 558 µm (Nguyen *et al.*, 2007). Velocity and acceleration were calculated as rate of change of distance and velocity with respect to time.
